# *Lactobacillus* Bacteremia and Endovascular Infections: A Retrospective Study of 100 Patients

**DOI:** 10.1093/ofid/ofaf466

**Published:** 2025-08-04

**Authors:** Christopher V Radcliffe, Melis N Anahtar, Elizabeth L Hohmann, Sarah E Turbett, Caitlin M Dugdale, Molly L Paras

**Affiliations:** Department of Medicine, Massachusetts General Hospital, Boston, Massachusetts, USA; Department of Pathology, Massachusetts General Hospital, Boston, Massachusetts, USA; Division of Infectious Diseases, Department of Medicine, Massachusetts General Hospital, Boston, Massachusetts, USA; Department of Medicine, Massachusetts General Hospital, Boston, Massachusetts, USA; Department of Pathology, Massachusetts General Hospital, Boston, Massachusetts, USA; Division of Infectious Diseases, Department of Medicine, Massachusetts General Hospital, Boston, Massachusetts, USA; Division of Infectious Diseases, Department of Medicine, Massachusetts General Hospital, Boston, Massachusetts, USA; Division of Infectious Diseases, Department of Medicine, Massachusetts General Hospital, Boston, Massachusetts, USA

**Keywords:** *Lactobacillus*, endocarditis, bacteremia, immunocompromised

## Abstract

**Background:**

Lactobacilli are gastrointestinal commensals and may represent skin contamination when isolated in blood cultures. Although uncommon, *Lactobacillus* bloodstream infections and endocarditis have been reported.

**Methods:**

We conducted a retrospective cohort study of patients with *Lactobacillus* species bacteremia and/or endovascular infections over a 22-year period to identify potential predictors of possible or definite endocarditis using the modified Duke criteria. *Lactobacillus* growth in an initial blood culture set without growth in subsequent blood culture sets collected within 7 days was labeled as blood culture contamination (BCC), and these were excluded from the primary analysis. The primary outcome was the proportion of patients with possible or definite endocarditis. For all *Lactobacillus* isolates, we collected antimicrobial susceptibility data determined via broth microdilution methods according to Clinical and Laboratory Standards Institute guideline M45.

**Results:**

We identified 331 patients with blood and/or endovascular cultures positive for *Lactobacillus*, of whom 100 were included. The primary outcome of possible or definite endocarditis was identified in 29% (29/100) of patients included in the primary analysis. Both the presence of an intracardiac device and/or non-native valve (relative risk [RR], 8.57; 95% CI, 1.89–38.83) and injection drug use (RR, 13.47; 95% CI, 3.18–57.03) were predictors of possible or definite endocarditis. All-cause mortality at ≤90 days was 22% (21/94).

**Conclusions:**

Nearly 1 in 3 patients with *Lactobacillus* bacteremia had possible or definite endocarditis, though polymicrobial infection was common. Growth of *Lactobacillus* spp. in blood cultures should be considered potentially pathogenic and interpreted in the clinical context before the isolate is labelled as inconsequential.

## Teaser

This retrospective study of Lactobacillus bacteremia andendovascular infections identified high all-cause mortality in a patient populationwith significant medical comorbidities. Lactobacilli can be pathogenic and shouldbe interpreted in the broader clinical context before being labeled as contaminantor inconsequential.

Lactobacilli are gram-positive, facultatively anaerobic rods that are generally considered to be beneficial members of the human gastrointestinal and vaginal microbiomes. Lactic acid–producing *Lactobacillus* strains are marketed as probiotics and are commonly used in food manufacturing [1, 2]. Given their generally limited pathogenicity, it can be difficult to determine the clinical significance of the detection of *Lactobacillus* species even in normally sterile sites like blood. Clinical microbiology laboratories often consider *Lactobacillus* spp. to be a contaminant if isolated in a single blood culture set in a blood culture series [3]. While uncommon, the isolation of *Lactobacillus* spp. in blood cultures can be clinically significant, with several reports of true bloodstream infections and endocarditis due to lactobacilli [1, 4–8]. However, the genus is not included in the American Heart Association or European Society of Cardiology endocarditis guidelines [9, 10]. Risk factors for clinically significant infection include immunocompromised status, presence of central venous catheters, and a history of gastrointestinal disease [7, 8]. Aside from these risk factors, emerging data suggest a link between probiotic use and *Lactobacillus* bacteremia in the intensive care setting [11]. Given the rising prevalence of immunocompromised patients in the United States, it is likely that lactobacilli infections will become increasingly relevant to clinical practice [12].

Species-level variability in antimicrobial susceptibility profiles adds complexity to the treatment of *Lactobacillus* bacteremia and endovascular infections [1]. Antimicrobial susceptibility testing (AST) is not reflexively performed for *Lactobacillus* spp., as it is often isolated from polymicrobial infections with multiple other anaerobes; however, AST should be considered when *Lactobacillus* is predominant or pure in culture from sterile sites [13]. Clinical breakpoints for *Lactobacillus* spp. are provided by the Clinical and Laboratory Standards Institute (CLSI) but not the European Committee on Antimicrobial Susceptibility Testing, and *Lactobacillus* susceptibility testing cannot be performed on commonly used automated systems like the VITEK 2 (bioMerieux) and Phoenix (Becton Dickinson) instruments. Thus, antimicrobial therapy is often determined empirically. Lactobacilli are frequently resistant to vancomycin and metronidazole, and experts recommend the use of penicillin or aminopenicillins [1, 7]. Genetic diversity among *Lactobacillus* spp. makes susceptibility patterns challenging to predict, and more data on real-world *Lactobacillus* bloodstream infection treatment outcomes are needed to inform clinical practice [1, 8].

Several prior case series of *Lactobacillus* bacteremia in the United States, Finland, and Taiwan have described common comorbid conditions, antimicrobial susceptibility patterns, and mortality; however, there was limited representation of modern endovascular devices and injection drug use [14–17]. To better understand the contemporary landscape of *Lactobacillus* bacteremia and/or endovascular infections in the United States, we conducted a retrospective study of patients with *Lactobacillus* bacteremia and/or endovascular infections managed in the Mass General Brigham (MGB) health care system over >2 decades. We aimed to evaluate patient characteristics, AST patterns, and real-world clinical outcomes of individuals with *Lactobacillus* bacteremia and/or endovascular infections.

## METHODS

### Study Overview

To identify patients with *Lactobacillus* bacteremia and/or endovascular infection, we searched the MGB Research Patient Data Registry (RPDR) to identify all adult (age ≥18 years) patients who received care at 1 or more of 8 in-network hospitals in Massachusetts or New Hampshire between January 1, 2000, and September 1, 2022, and whose electronic medical record (EMR) contained the term “lactobacillus” [18]. We included all patients found to have growth of *Lactobacillus* spp. in cultures obtained from blood, mediastinum, vascular tissue, vascular graft materials, intravascular lines, intravascular devices, heart valves, pericardium, pericardial space, thrombi, and/or unspecified locations from this RDPR search for further review. In addition to the MGB RPDR, we queried the Massachusetts General Hospital (MGH) Clinical Microbiology Laboratory Information System (LIS) to verify inclusion of all adult patients with blood cultures demonstrating growth of *Lactobacillus* spp. during the study period. The MGB Institutional Review Board reviewed and approved the study protocol (#2022P002417).

### Data Collection

Authors C.V.R., C.M.D., and M.L.P. performed manual chart review of the EMR. The MGB system transitioned to Epic (Verona, WI, USA) in April 2016, so patients' EMRs before this transition were reviewed using the pre-Epic system known as Partners Healthcare System (PHS) viewer. Clinical documentation during the PHS viewer era involved use of physical charts, and EMRs on PHS viewer do not routinely have complete documentation available. Cases flagged by 1 of these authors as requiring adjudication were discussed between 2 authors, and data were entered into a standardized REDCap form. We recorded patient demographics, comorbidities, and possible risk factors for *Lactobacillus* infection [14–17]. The MGB definition of immunocompromised status was applied to each patient (Supplementary Data). In addition to antibiotic therapy, we noted surgical and/or procedural interventions performed for source control.

We obtained diagnostic information from the EMR and MGH LIS. When echocardiography was obtained after the first positive culture, we applied the 2000 Duke criteria for infective endocarditis, as the 2023 revised edition was not available at the time of this study's inception [19, 20]. For patients with positive blood cultures, we applied a study definition of blood culture contamination (BCC) adapted from the MGH microbiology laboratory definition. We labeled all patients with *Lactobacillus* growth in an initial blood culture set without growth of *Lactobacillus* in subsequent blood culture sets collected within 7 days as cases of BCC. To avoid overestimation of the BCC rate, we included patients with *Lactobacillus* growth in an initial blood culture set without repeat blood culture sets obtained within 7 days in our primary analysis. We excluded patients with BCC from the study's primary analysis but included AST data from their *Lactobacillus* isolates. For *Lactobacillus* spp. isolates with AST data available, we recorded minimum inhibitory concentrations (MICs) obtained using broth microdilution methods and applied breakpoints from CLSI M45 [13].

We summarized the identity and duration of antibiotic therapy with activity against lactobacilli. If no AST data were available, we assumed amoxicillin ± clavulanate, ampicillin ± sulbactam, daptomycin, linezolid, and penicillin to cover lactobacilli based on extrapolation of CLSI M45 guidelines [13]. We also assumed piperacillin-tazobactam to cover lactobacilli based on susceptibility to penicillin and prior report of low MICs obtained with gradient diffusion methods [16]. We recorded both empiric and targeted therapy. We defined empiric therapy as the use of an antibiotic with anti-*Lactobacillus* activity without explicit documentation of intent to treat the *Lactobacillus* isolate(s). We defined targeted therapy as a course of an anti-*Lactobacillus* antibiotic with documented intention to treat the *Lactobacillus* isolate(s). For patients who received targeted therapy, we recorded the identity of the intended treatment regimen. The primary outcome of our study was the proportion of patients with possible or definite endocarditis. For secondary outcomes, we report all-cause mortality at ≤90 days and ≤365 days and recurrent *Lactobacillus* bacteremia ≤90 days after initial diagnosis.

### Statistical Analyses

We generated summary statistics for all components of the analysis. We identified potential predictors of possible or definite endocarditis (as compared with rejected endocarditis) using the modified Duke criteria among individuals with positive *Lactobacillus* blood and/or endovascular cultures not meeting criteria for BCC using complete case analysis with a Fisher exact test for comparison or a Kruskal-Wallis test where appropriate. Data imputation was not performed, and the proportion of missing data was reported for each variable. We analyzed individuals without echocardiography available in combination with those for whom endocarditis was rejected by the Duke criteria. *P* < .05 was considered statistically significant. We conducted statistical analyses in Stata, version 17 (College Station, TX, USA).

## RESULTS

### Study Population and Characteristics

**Figure 1.**
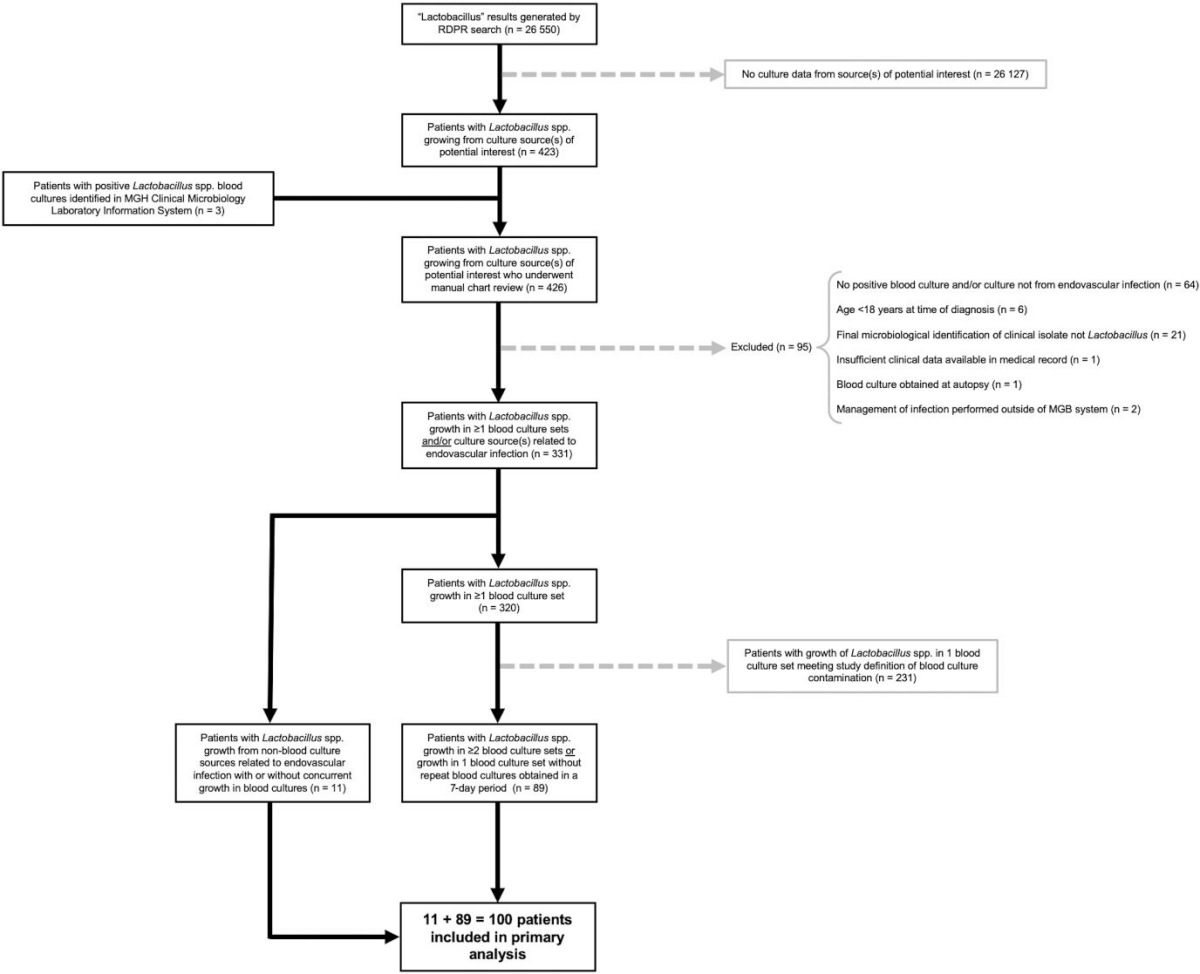
Summary of the inclusion process for patients with *Lactobacillus* bacteremia and/or endovascular infections in the primary analysis. Patients with culture sources of potential interest had growth of *Lactobacillus* spp. in cultures obtained from blood, mediastinum, vascular tissue, vascular graft materials, intravascular lines, intravascular devices, heart valves, pericardium, pericardial space, thrombi, and/or unspecified locations. Abbreviations: MGB, Mass General Brigham; MGH, Massachusetts General Hospital; RDPR, Research Patient Data Registry.

The database search led to identification of 331 patients with growth of *Lactobacillus* in blood cultures and/or culture sources related to endovascular infection in the MGB system between January 2000 and September 2022 (Figure 1). Two hundred thirty-one patients met the study definition of BCC and were thus excluded from the primary analysis. No statistically significant differences in demographics or clinical characteristics were identified between patients with BCC and patients included in the primary analysis (Supplementary Table 1).

For the 100 patients included in the primary analysis, the median age (interquartile range) was 58 (39–69) years, and 47% (47/100) were female (Table 1). Patients with rejected endocarditis were older than patients with possible or definite endocarditis (*P* < .01). Diabetes mellitus had been diagnosed in 24% (24/100), and 37% (37/100) of patients were immunocompromised. The most common immunocompromising conditions were cytotoxic chemotherapy within the prior 3 months (17/37; 46%), active leukemia or lymphoma (12/37; 32%), and metastatic cancer (7/37; 19%). Indwelling lines at time of the first positive culture were common (62/97; 64%). Thirty-two (32/100; 32%) patients had 1 or more types of foreign hardware, and endovascular (14/32; 44%) or orthopedic (10/32; 31%) hardware was most frequently present.

**Table 1.** Patient Characteristics and Risk Factors for Possible or Definite Endocarditis

	Total (n = 100)	Rejected Endocarditis^a^ (n = 71)	Possible or Definite Endocarditis (n = 29)	Relative Risk (95% CI)	*P* Value
Age, median (IQR), y	58 (39–69)	62 (44–70)	45 (30–55)	Not applicable	<.01
Female, No. (%)	47/100 (47)	32/71 (45)	15/29 (52)	1.15 (0.74–1.77)	.66
Diabetes mellitus, No. (%)	24/100 (24)	18/71 (25)	6/29 (21)	0.82 (0.36–1.85)	.80
Inflammatory bowel disease, No. (%)	5/100 (5)	4/71 (6)	1/29 (3)	0.61 (0.07–5.25)	1.00
Liver disease, No. (%)	28/100 (28)	17/71 (24)	11/29 (38)	1.58 (0.85–2.95)	.22
End-stage renal disease requiring dialysis, No. (%)	6/100 (6)	4/71 (6)	2/29 (7)	1.22 (0.24–6.32)	1.00
Immunocompromised, No. (%)	37/100 (37)	31/71 (44)	6/29 (21)	0.47 (0.22–1.01)	.04
Injection drug use, No. (%)	13/100 (13)	2/71 (3)	11/29 (38)	13.47 (3.18–57.03)	<.01
Indwelling line, No. (%)	62/97 (64)	44/71 (62)	18/26 (69)	1.12 (0.82–1.53)	.64
Intracardiac device and/or non-native valve, No. (%)	9/100 (9)	2/71 (3)	7/29 (24)	8.57 (1.89–38.83)	<.01
Intraperitoneal surgery and/or gastrointestinal endoscopy ≤30 d before diagnosis, No. (%)	29/100 (29)	25/71 (35)	4/29 (14)	0.39 (0.15–1.03)	.05
*Clostridioides difficile* infection ≤14 d before diagnosis, No. (%)	3/100 (3)	2/71 (3)	1/29 (3)	1.22 (0.12–12.98)	1.00
Use of total or partial parenteral nutrition ≤14 d before diagnosis, No. (%)	24/100 (24)	16/71 (23)	8/29 (28)	1.22 (0.59–2.54)	.61
≥1 additional organism(s) isolated in culture(s) that grew *Lactobacillus* species, No. (%)	43/100 (43)	27/71 (38)	16/29 (55)	1.45 (0.93–2.26)	.13

Denominators reflect the number of patients for whom there were sufficient data to assess the characteristic.

Abbreviation: IQR, interquartile range.

^a^Rejected endocarditis category includes cases for which Duke criteria were unable to be applied due to absence of echocardiography.

Patients with possible or definite endocarditis were less likely to be immunocompromised when compared with those with rejected endocarditis (*P* = .04). Gastrointestinal endoscopy and/or intraperitoneal surgery ≤30 days before the first positive culture occurred in 29% (29/100) of patients, and the difference in the proportion of patients who underwent these procedures approached statistical significance (*P* = .05). An intracardiac device and/or non-native valve was present in 9 of 100 (9%) patients, with bioprosthetic valves being most common (4/9; 44%). Injection drug use was reported for 13/100 patients (13%). Both the presence of an intracardiac device and/or non-native valve (relative risk [RR], 8.57; 95% CI, 1.89–38.83) and injection drug use (RR, 13.47; 95% CI, 3.18–57.03) increased the relative risk of possible or definite endocarditis.

Echocardiography was performed for 66 of 100 patients (66%). Most patients with echocardiography available were classified as rejected endocarditis (37/66; 56%). Possible and definite endocarditis were identified in 21% (21/100) and 8% (8/100) patients, respectively. Echocardiography identified valvular vegetations and new valvular regurgitation in 5/66 (8%) and 3/66 (5%) patients, respectively. Clinically recognized embolic phenomena affected 52% (14/27) of patients with possible or definite endocarditis.

### Microbiology and Antimicrobial Susceptibility Data

Most patients had growth of lactobacilli in ≥2 sets of blood cultures (71/100; 71%), and 18 patients (18/100; 18%) had growth of lactobacilli in 1 blood culture set without repeat blood culture sets drawn within 7 days. The remaining 11 patients had growth of lactobacilli in cultures obtained from the vessel wall or vascular graft material (8/100; 8%), indwelling lines (2/100; 2%), or an implanted cardioverter-defibrillator device (1/100; 1%). The growth of ≥1 additional organism(s) in cultures growing lactobacilli was common (43/100; 43%). Of the 331 patients with *Lactobacillus* isolates, 68 unique isolates had AST data determined with broth microdilution methods. All isolates were susceptible to ampicillin, penicillin, clindamycin, daptomycin, erythromycin, and linezolid (Table 2). Most isolates were resistant to meropenem (33/40; 83%) and vancomycin (65/67; 97%).

**Table 2.** Antimicrobial Susceptibility Data for Unique *Lactobacillus* Isolates

Antimicrobial	No. of Unique Isolates	MIC Range, µg/mL	Categorical Interpretation of MIC		
			Susceptible, No. (%)	Intermediate, No. (%)	Resistant, No. (%)
Penicillin	68	≤0.06 to 8	68 (100)	0	0
Ampicillin	68	≤0.12 to 8	68 (100)	0	0
Imipenem	55	≤0.5 to 2	11 (20)	29 (53)	15 (27)
Meropenem	40	0.25 to >8	6 (15)	1 (3)	33 (83)
Vancomycin	67	<0.25 to >32	2 (3)	0	65 (97)
Daptomycin	31	<0.5 to 2	31 (100)	0	0
Erythromycin	67	<0.25 to ≤0.5	67 (100)	0	0
Clindamycin	67	<0.5 to ≤0.5	67 (100)	0	0
Linezolid	64	<1 to 4	64 (100)	0	0

Minimum inhibitory concentration values were obtained using broth microdilution methods and applied breakpoints from the CLSI M45 [13]. One isolate's MIC for imipenem was reported as ≤2 µg/mL and excluded from the above summary due to inability to apply CLSI breakpoints. See Supplementary Table 2 for individualized information on each of the 68 isolates.

Abbreviations: CLSI, Clinical Laboratory Standards Institute; MIC, minimum inhibitory concentration.

### Management and Outcomes

Management approaches for *Lactobacillus* spp. bacteremia and/or endovascular infections are summarized in Table 3. For the 97 patients with antibiotic treatment information available, 25% (24/97) did not receive an antibiotic with activity against lactobacilli. Empiric antibiotic therapy was administered in 18% (13/73) of patients who received antibiotics with activity against lactobacilli. For the 56 patients who received targeted therapy and had information on treatment duration available, 68% (38/56) had an intended duration of <6 weeks, and 32% (18/56) ≥6 weeks. The most common agents used in targeted therapy regimens were penicillin (17/60; 28%), ampicillin (12/60; 20%), and piperacillin-tazobactam (12/60; 20%). For patients with an indwelling line and information available, the line was removed in most cases (42/54; 78%). Noncardiac surgical and/or procedural interventions were performed for source control in 28% (28/100) of patients. Procedures related to intra-abdominal sources with or without extension to other sites were most common (19/28; 68%), and vascular intervention occurred in 8 patients (8/28; 29%).

**Table 3.** Treatment Strategies and Outcomes for *Lactobacillus* Bacteremia and/or Endovascular Infections

	Total (n = 100), No. (%)	Rejected Endocarditis^a^ (n = 71), No. (%)	Possible or Definite Endocarditis (n = 29), No. (%)	*P* Value
Anti-*Lactobacillus* antibiotic(s) administered				.01
Yes	73/97 (75)	47/69 (68)	26/28 (93)	
No	24/97 (25)	22/69 (32)	2/28 (7)	
Received *Lactobacillus*-targeted therapy (rather than empiric therapy only)	60/73 (82)	39/47 (83)	21/26 (81)	1.00
Surgical and/or procedural intervention(s) performed for source control	30/100 (30)	23/71 (32)	7/29 (24)	.48
Recurrent *Lactobacillus* bacteremia ≤90 d after initial episode	1/94 (1)	0/66 (0)	1/28 (4)	.30
All-cause mortality ≤90 d after diagnosis	21/94 (22)	17/66 (26)	4/28 (14)	.29
All-cause mortality ≤365 d after diagnosis	35/90 (39)	29/64 (45)	6/26 (23)	.06

Denominators reflect the number of patients for whom there were sufficient data to assess the characteristic.

^a^Rejected endocarditis category includes cases for which Duke criteria were unable to be applied due to absence of echocardiography.

Three patients (3/100; 3%) underwent cardiac surgery. One patient had definite prosthetic valve endocarditis and underwent redo aortic valve replacement, with bacterial rods seen with silver staining of the resected prosthetic valve. The remaining 2 patients had possible endocarditis upon initial presentation but underwent cardiac surgery in subsequent months. One patient underwent elective mitral valve repair 1 year after *Lactobacillus* bacteremia, and pathology identified endocarditis with gram-variable organisms having bacillary morphology on hematoxylin and eosin and methenamine silver stains. 16S ribosomal deoxyribonucleic acid sequencing performed on posterior mitral valve tissue identified *Lactobacillus rhamnosus*. The third patient who underwent cardiac surgery was the only patient (1/94; 1%) who had recurrent *Lactobacillus* bacteremia ≤90 days after initial diagnosis. Prosthetic valve endocarditis with aortic root abscess was diagnosed during the second episode, and redo aortic valve replacement with aortic root repair was performed. Pathology identified endocarditis with bacterial rods on Steiner stain.

For patients with follow-up available, all-cause mortality at ≤90 days and ≤365 days was 22% (21/94) and 39% (35/90), respectively. Patients with rejected endocarditis had higher all-cause mortality at ≤90 and ≤365 days when compared with patients with possible or definite endocarditis, but the differences in all-cause mortality were not statistically significant. Table 4 summarizes individualized treatment and outcomes for patients with definite endocarditis.

**Table 4.** Summary of Definite Endocarditis Cases

Age, y/Gender	Relevant Comorbidities and/or Risk Factors	*Lactobacillus* Isolate(s)	Concurrent Pathogens	Suspected Source	Echocardiographic Findings	Embolic Phenomena	*Lactobacillus*-Active Antibiotic Agent(s)	Surgical Intervention	Outcome
74/female	TAVR	*Lactobacillus* species	None	Unknown	New aortic perivalvular leak and thickened aortic valve leaflets	Splenic infarct	IV penicillin and gentamicin	After failure of medical therapy, redo aortic valve replacement, explant of TAVR, and aortic root enlargement (bovine pericardial patch)	Death ≤365 d after diagnosis
50/male	Injection drug use	*Lactobacillus* species, *Lactobacillus paracasei*	None	Injection drug use	Multiple masses of echoes on coronary cusps of aortic valve (largest 20 × 15 × 13 mm) and focal thickening of aortic root suggestive of early aortic abscess	Possible cerebral microhemorrhages	IV penicillin	None	<90 d of follow-up
54/female	Tunneled CVC on TPN, recent *C. difficile* infection	*Lactobacillus* species	None	Abdominal translocation vs TPN/line-related	8 × 6-mm thrombus or vegetation on Eustachian valve	Septic pulmonary emboli	IV penicillin	None	Death ≤90 d after diagnosis
55/male	Infliximab use for sarcoidosis, CVC, recent repair of atrio-esophageal fistula, recent parenteral nutrition	*Lactobacillus* species, *Lactobacillus acidophilus*	Pericardial fluid culture with viridans streptococci	Atrio-esophageal fistula	Large mobile mass in the left atrium consistent with thrombus ≥25 mm	None	IV penicillin and gentamicin	None	Alive 365 d after diagnosis
45/female	Injection drug use, diabetes mellitus	*Lactobacillus* species	Blood cultures with *Enterococcus faecalis*	Injection drug use	Possible echodensity near the right coronary leaflet seen on TTE but not TEE	Splenic abscess	IV ampicillin	Percutaneous drainage of splenic abscess	Alive 365 d after diagnosis
29/male	Injection drug use, chronic HCV infection	*Lactobacillus* species	Incompletely treated *Staphylococcus aureus* and *Streptococcus mitis* endocarditis in recent months	Injection drug use	Mass of echoes attached to aortic valve consistent with vegetation (12 × 5 mm) and severe aortic regurgitation; increased mitral valve thickening	Cerebral embolus	IV daptomycin	None	<365 d of follow-up
26/female	Injection drug use, PICC	*Lactobacillus* species	Blood cultures with *Candida albicans, Candida dubliniensis, Capnocytophaga* spp., *Neisseria* spp., *S. aureus*	Injection drug use	No new relevant findings on repeat echocardiography performed after blood cultures newly positive for *Lactobacillus* spp.	Orbital abscess and septic pulmonary emboli	Ampicillin (IV and per os formulations used)	None	<365 d of follow-up
82/male	Bioprosthetic aortic valve, cirrhosis	*Lactobacillus rhamnosus*	Blood cultures with *Staphylococcus hominis*	Unknown; query dental source	Newly thickened aortic valve leaflets	Renal and splenic infarcts	IV penicillin	None	Alive 365 d after diagnosis

Abbreviations: CVC, central venous catheter; HCV, hepatitis C virus; IV, intravenous; PICC, peripherally inserted central catheter; TAVR, transcatheter aortic valve repair; TEE, transesophageal echocardiogram; TPN, total parenteral nutrition; TTE, transthoracic echocardiogram.

## DISCUSSION

We performed a retrospective cohort study of 100 patients with *Lactobacillus* bacteremia and/or endovascular infection managed in the MGB system. Diabetes mellitus, liver disease, immunocompromised status, and presence of indwelling lines and/or hardware were common. Roughly 40% of episodes involved >1 pathogen, and definite or probable endocarditis affected 30% of patients with growth of *Lactobacillus* from blood cultures. Injection drug use and presence of intracardiac device and/or non-native valve were most predictive of possible or definite endocarditis. Among individuals with definite or possible endocarditis, 10% underwent cardiac surgery. All-cause mortality in the setting of *Lactobacillus* bacteremia and/or endovascular infection was high with or without endocarditis.

The isolation of lactobacilli in blood cultures or cultures related to endovascular sources must be interpreted with caution. The clinical relevance assigned to a *Lactobacillus* isolate in a blood culture set ought to account for the host's net state of immunosuppression, severity of illness, presence of co-pathogens, and plausibility of a source. Similar to prior reports, our data implicate lactobacilli as potential pathogens in patients with conditions such as malignancy, liver disease, immunocompromised status, and abdominal surgery [8, 14, 15, 17]. To assist in the interpretation of *Lactobacillus* spp., it is best to obtain at least 2 sets of blood cultures from different sites to help differentiate BCC from potential pathogen.

In addition to the above conditions, we report a comparatively high prevalence (13%) of injection drug use. For patients with injection drug use and definite endocarditis, co-pathogens like *Staphylococcus aureus* and *Enterococcus faecalis* likely contributed to the observed valvular pathology. *Lactobacillus* infections are rarely associated with injection drug use, but they are recognized as one of several atypical bacterial pathogens affecting people who inject drugs [21–24]. A recent study identified gut microbiome changes in the context of injection drug use for people living with or without HIV and reported that certain genera including *Lactobacillus* were over-represented in people who inject multiple drugs [25]. Such alterations in microbiota may have clinical implications for the microbiology of bloodstream infections in this population.

Elevated all-cause mortality at ≤90 days (22%) and ≤365 days (39%) was noted in our study population, and similar observations have been reported [14, 15, 17, 26]. With substantial comorbidities often preceding or accompanying *Lactobacillus* bacteremia, 1-month mortality has ranged from 26% to 42% in other published series [14, 17]. Some authors label *Lactobacillus* bacteremia as a negative prognostic sign or marker of severe underlying disease [15, 26, 27]. These findings are interesting to note as lactobacilli are often viewed as low-virulence pathogens. In comparison, *S. aureus* bacteremia all-cause mortality at 3 months and 1 year was reported as 27% and 30%, respectively, in a recent meta-analysis [28]. Co-pathogens are important to consider when accounting for all-cause mortality of *Lactobacillus* bacteremia. Concordant with prior reports, our study identified 43% of patients to have ≥1 organism isolated in cultures growing lactobacilli [14, 15, 17]. Both co-pathogens and comorbidities like immunocompromised status and end-organ dysfunction may account for a significant proportion of the all-cause mortality.

We paradoxically report higher all-cause mortality in patients with rejected endocarditis compared with patients with possible or definite endocarditis. This finding may be explained by the rejected endocarditis group's older age (*P* < .01) and greater proportion of immunocompromised patients (44% vs 21%; *P* = .04). For example, 6/7 (86%) patients with metastatic cancer and 11/12 (92%) patients with active leukemia or lymphoma belonged to the rejected endocarditis group. Notably, we could not apply Duke criteria to patients who died without echocardiography and were resultantly grouped with rejected endocarditis cases. Therefore, our results may be subject to immortal time bias, which could overestimate the all-cause mortality of rejected endocarditis. Separate from comorbid conditions, fewer patients (68% vs 93%; *P* = .01) with rejected endocarditis received antibiotics with activity against lactobacilli. Salminen et al. previously reported lower mortality when effective antibiotics were used for infections caused by lactobacilli, but the results were not statistically significant [16]. Further studies are needed to explore whether targeted treatment of lactobacilli impacts all-cause or infection-related mortality.

Our study has several limitations. First, our study has limitations inherent to the retrospective design, and collection of standardized, prospective data was not possible. As such, some clinical variables were unable to be gleaned from the EMR. Specifically, the hospital system's pre-Epic EMR system was used to record heterogeneous data on patients with hospital encounters before April 2016, and the level of granularity was often insufficient to record all intended data points. To account for this limitation and avoid undue emphasis on the isolation of lactobacilli, we applied a BCC definition to all patients identified in our database search and excluded cases of BCC from our primary analysis. Second, this study was conducted across a single health care system. While there are multiple acute care hospitals across this system, practice patterns regarding the care of patients with *Lactobacillus* bacteremia may differ across hospitals. Third, most *Lactobacillus* isolates in our study were identified to the genus level, so we are unable to comment on species-specific differences in pathogenicity, antimicrobial susceptibility patterns, and outcomes. Finally, due to our limited sample size, we were unable to adjust for possible confounders when calculating relative risks.

Overall, our retrospective study of 100 patients with *Lactobacillus* bacteremia and/or endovascular infection identified definite or probable endocarditis in one-third of patients, and co-pathogens were common. Consistent with prior reports, all-cause mortality was high. Given the rising prevalence of immunocompromised patients and older adults, the identification of lactobacilli in blood or endovascular cultures should be cautiously interpreted in patients with clinical pictures concerning for bloodstream or endovascular infection. Further studies are needed to explore the effects of targeted therapy on outcomes and determine the relative contribution of *Lactobacillus* infections to all-cause mortality.

## Supplementary Material

ofaf466_Supplementary_Data

## Supplementary Data

Supplementary materials are available at *Open Forum Infectious Diseases* online. Consisting of data provided by the authors to benefit the reader, the posted materials are not copyedited and are the sole responsibility of the authors, so questions or comments should be addressed to the corresponding author.
