# Supplementary material for: Lactobacillus Bacteremia and Endovascular Infections: A Retrospective Study of 100 Patients
Source: Open Forum Infect Dis. 2025 Aug 4;12(8):ofaf466. doi: 10.1093/ofid/ofaf466 (PMC12368416; doi:10.1093/ofid/ofaf466)
Supplement: ofaf466_Supplementary_Data [file ofaf466_supplementary_data.docx]

**TABLE OF CONTENTS**

**Supplement 1:** Definition of immunocompromised **pg. 1**

**Table 1:** Demographics and clinical characteristics of patients with blood culture

contamination and patients included in the primary analysis **pg. 2**

**Table 2:** Minimum inhibitory concentrations (MICs) and categorical interpretations

for each unique *Lactobacillus* spp. isolate **pg. 3**

**Supplement 2:** STROBE checklist for cohort studies **pg. 6**

**References**  **pg. 8**

**Supplement 1:** Definition of immunocompromised

Mass General Brigham criteria for immunocompromised status were applied to patients in the study. Patients were labelled as having immunocompromised status based on one or more of the following diagnoses:

- 1. Human immunodeficiency virus infection with CD4 <200/mm^3^
  2. Active lymphoma or leukemia (including indolent chronic lymphocytic leukemia)
  3. Metastatic cancer
  4. Cytotoxic chemotherapy within the prior 3 months
  5. Radiation therapy within prior 3 months
  6. Congenital immunodeficiency
  7. Aplastic anemia
  8. Solid organ transplant recipients on immunosuppressive therapy
  9. Hematopoietic stem cell transplant recipients, unless >2 years post-transplant and no longer on any immunosuppressive therapy
  10. Immunocompromised based on ≥1 of the following medications: glucocorticoid therapy equivalent to prednisone ≥20 mg/d for ≥2 weeks, or if such therapy has been discontinued within the past month; alkylating agents within the past 3 months; antimetabolites (methotrexate >0.4 mg/kg/week, azathioprine >3 mg/kg/day, 6-mercaptopurine >1.5 mg/kg/day) within the past 3 months; cyclosporine, tacrolimus, sirolimus, everolimus, or mycophenolate mofetil within the past 3 months; biologic immunosuppressants and immunomodulators within the past 3 months (6 months for lymphocyte-depleting agents)

**Table 1**: Demographics and clinical characteristics of patients with blood culture

contamination and patients included in the primary analysis

|  | **Patients with blood culture contamination (n = 231)** | **Patients included in primary analysis (n = 100)** | **Total (n = 331)** | **p-value** |
| --- | --- | --- | --- | --- |
| **Age (median, [interquartile range])** | 59 [40, 70] | 58 [39, 69] | 58 [39, 70] | 0.49 |
| **Female (No., [%])** | 93/231 [40%] | 47/100 [47%] | 140/331 [42%] | 0.28 |
| **Diabetes mellitus (No., [%])** | 59/230 [26%] | 24/100 [24%] | 83/330 [25%] | 0.78 |
| **Inflammatory bowel disease (No., [%])** | 7/231 [3%] | 5/100 [5%] | 12/331 [4%] | 0.36 |
| **Liver disease (No., [%])** | 69/231 [30%] | 28/100 [28%] | 97/331 [29%] | 0.79 |
| **End stage renal disease requiring dialysis (No., [%])** | 9/231 [4%] | 6/100 [6%] | 15/331 [5%] | 0.40 |
| **Immunocompromised (No., [%])** | 81/230 [35%] | 37/100 [37%] | 118/330 [36%] | 0.80 |
| **Injection drug use (No., [%])** | 24/231 [10%] | 13/100 [13%] | 37/331 [11%] | 0.57 |
| **Indwelling line (No., [%])** | 145/225 [64%] | 62/97 [64%] | 207/322 [64%] | 1.00 |
| **Intracardiac device and/or non-native valve (No., [%])** | 26/231 [11%] | 9/100 [9%] | 35/331 [11%] | 0.70 |
| **Intraperitoneal surgery and/or gastrointestinal endoscopy ≤30 days before diagnosis (No., [%])** | 65/231 [28%] | 29/100 [29%] | 94/331 [28%] | 0.90 |
| ***Clostridioides difficile* infection ≤14 days before diagnosis (No., [%])** | 11/231 [5%] | 3/100 [3%] | 14/331 [4%] | 0.57 |
| **Use of total or partial parenteral nutrition ≤14 days before diagnosis (No., [%])** | 36/225 [16%] | 24/100 [24%] | 60/325 [18%] | 0.09 |
| **≥1 additional organism(s) isolated in culture(s) which grew *Lactobacillus* species (No., [%])** | 78/231 [34%] | 43/100 [43%] | 121/331 [37%] | 0.14 |

Denominators reflect number of patients for whom there were sufficient data to assess the characteristic.

**Supplement Table 2:** Minimum inhibitory concentrations (MICs) and categorical interpretations for each unique *Lactobacillus* spp. isolate

|  | Penicillin  (n = 68) | | Ampicillin  (n = 68) | | Imipenem  (n = 56) | | Meropenem  (n = 40) | | Clindamycin  (n = 67) | | Daptomycin  (n = 31) | | Erythromycin (n = 67) | | Linezolid  (n = 64) | | Vancomycin  (n = 67) | |
| --- | --- | --- | --- | --- | --- | --- | --- | --- | --- | --- | --- | --- | --- | --- | --- | --- | --- | --- |
| *Lactobacillus* spp. isolate number | MIC (µg/ mL) | Interp. | MIC (µg/ mL) | Interp. | MIC (µg/ mL) | Interp. | MIC (µg/ mL) | Interp. | MIC (µg/ mL) | Interp. | MIC (µg/ mL) | Interp. | MIC (µg/ mL) | Interp. | MIC (µg/mL) | Interp. | MIC (µg/ mL) | Interp. |
| 1 | 2 | S | 8 | S | ≤0.5 | S | ≤0.5 | S | ≤0.5 | S |  |  | ≤0.5 | S | ≤1 | S | >16 | R |
| 2 | 1 | S | 2 | S | 1 | I | 8 | R | ≤0.5 | S |  |  | ≤0.5 | S | ≤1 | S | >16 | R |
| 3 | 0.5 | S | 1 | S | 2 | R | 8 | R | ≤0.5 | S |  |  | ≤0.5 | S | ≤1 | S | >16 | R |
| 4 | 4 | S | 4 | S | 2 | R | >8 | R | ≤0.5 | S |  |  | ≤0.5 | S | 2 | S | >16 | R |
| 5 | 1 | S | 2 | S | 2 | R |  |  | ≤0.5 | S | ≤0.5 | S | ≤0.5 | S | ≤1 | S | >16 | R |
| 6 | 0.5 | S | 1 | S | 1 | I | 4 | R | ≤0.5 | S |  |  | ≤0.5 | S | ≤1 | S | >16 | R |
| 7 | 2 | S | 2 | S | 2 | R |  |  | ≤0.5 | S | ≤0.5 | S | ≤0.5 | S | ≤1 | S | >16 | R |
| 8 | 0.5 | S | 0.5 | S |  |  |  |  |  |  |  |  |  |  |  |  |  |  |
| 9 | 1 | S | 1 | S | 1 | I |  |  | ≤0.5 | S | 1 | S | ≤0.5 | S | 2 | S | >16 | R |
| 10 | 0.5 | S | 1 | S |  |  |  |  | ≤0.5 | S | ≤0.5 | S | ≤0.5 | S | 2 | S | >16 | R |
| 11 | ≤0.06 | S | ≤0.12 | S | ≤0.5 | S |  |  | ≤0.5 | S | 1 | S | ≤0.25 | S | 2 | S | <0.25 | S |
| 12 | 1 | S | 1 | S | 2 | R |  |  | ≤0.5 | S | 1 | S | ≤0.25 | S | ≤1 | S | >32 | R |
| 13 | 1 | S | 1 | S | 1 | I | 8 | R | ≤0.5 | S |  |  | ≤0.5 | S | ≤1 | S | >16 | R |
| 14 | 0.5 | S | 1 | S | 1 | I |  |  | ≤0.5 | S | ≤0.5 | S | ≤0.5 | S | ≤1 | S | >16 | R |
| 15 | 1 | S | 0.5 | S | ≤0.5 | I |  |  | ≤0.5 | S | ≤0.5 | S | ≤0.5 | S | ≤1 | S | >16 | R |
| 16 | 1 | S | 2 | S | 1 | I |  |  | ≤0.5 | S | ≤0.5 | S | ≤0.5 | S | ≤1 | S | >16 | R |
| 17 | 4 | S | 4 | S | 2 | R | >8 | R | ≤0.5 | S |  |  | ≤0.5 | S | 2 | S | >16 | R |
| 18 | 1 | S | 2 | S |  |  |  |  | ≤0.5 | S |  |  | ≤0.5 | S | 2 | S | >16 | R |
| 19 | 1 | S | 1 | S | 2 | R | 8 | R | ≤0.5 | S |  |  | ≤0.5 | S | ≤1 | S | >16 | R |
| 20 | 1 | S | 1 | S | 2 | R | >8 | R | ≤0.5 | S |  |  | ≤0.5 | S | ≤1 | S | >16 | R |
| 21 | 1 | S | 1 | S |  |  | 8 | R | ≤0.5 | S | 1 | S | ≤0.25 | S | ≤1 | S | >32 | R |
| 22 | 1 | S | 2 | S |  |  | >8 | R | ≤0.5 | S | 2 | S | ≤0.25 | S | ≤1 | S | >32 | R |
| 23 | 1 | S | 1 | S | 1 | I |  |  | ≤0.5 | S | 1 | S | ≤0.5 | S | 2 | S | >16 | R |
| 24 | 0.5 | S | 1 | S | 1 | I |  |  | <0.5 | S | <0.5 | S | <0.25 | S | <1 | S | ≥32 | R |
| 25 | 0.25 | S | 0.25 | S |  |  | 0.25 | S | ≤0.5 | S | ≤0.5 | S | ≤0.25 | S | ≤1 | S | >32 | R |
| 26 | 0.5 | S | 1 | S | 1 | I | 8 | R | ≤0.5 | S | ≤0.5 | S | ≤0.5 | S | 2 | S | >16 | R |
| 27 | 1 | S | 2 | S | 1 | I | 8 | R | ≤0.5 | S |  |  | ≤0.5 | S | 2 | S | >16 | R |
| 28 | 1 | S | 2 | S | 2 | R | 8 | R | ≤0.5 | S |  |  | ≤0.5 | S | ≤1 | S | >16 | R |
| 29 | 1 | S | 2 | S | 1 | I |  |  | ≤0.5 | S | ≤0.5 | S | ≤0.5 | S | 2 | S | >16 | R |
| 30 | 0.5 | S | 1 | S |  |  | 2 | I | ≤0.5 | S | ≤0.5 | S | ≤0.25 | S | ≤1 | S | >32 | R |
| 31 | 0.5 | S | 1 | S | 1 | I | 8 | R | ≤0.5 | S |  |  | ≤0.5 | S | ≤1 | S | >16 | R |
| 32 | 1 | S | 1 | S | 1 | I | 4 | R | ≤0.5 | S |  |  | ≤0.5 | S | ≤1 | S | >16 | R |
| 33 | 0.5 | S | 1 | S | ≤0.5 | S | 4 | R | ≤0.5 | S |  |  | ≤0.5 | S | ≤1 | S | >16 | R |
| 34 | 0.5 | S | 1 | S | 1 | I | 8 | R | ≤0.5 | S |  |  | ≤0.5 | S | 2 | S | >16 | R |
| 35 | ≤0.12 | S | ≤0.25 | S | ≤0.5 | S | ≤0.5 | S | ≤0.5 | S |  |  | ≤0.5 | S | ≤1 | S | >16 | R |
| 36 | 2 | S | 2 | S |  |  |  |  | ≤0.5 | S |  |  | ≤0.25 | S |  |  | >32 | R |
| 37 | 1 | S | 1 | S | 1 | I |  |  | ≤0.5 | S | ≤0.5 | S | ≤0.5 | S | ≤1 | S | >16 | R |
| 38 | 1 | S | 0.5 | S |  |  |  |  | ≤0.5 | S | ≤0.5 | S | ≤0.5 | S | ≤1 | S | >16 | R |
| 39 | 4 | S | 4 | S | 2 | R |  |  | ≤0.5 | S | 1 | S | ≤0.5 | S | 2 | S | >16 | R |
| 40 | 1 | S | 2 | S | 1 | I |  |  | ≤0.5 | S | ≤0.5 | S | ≤0.25 | S | ≤1 | S | >32 | R |
| 41 | 0.5 | S | ≤0.25 | S | ≤0.5 | S |  |  | ≤0.5 | S | ≤0.5 | S | ≤0.5 | S | 2 | S | >16 | R |
| 42 | 8 | S | 2 | S | ≤0.5 | S |  |  | ≤0.5 | S | ≤0.5 | S | ≤0.5 | S | 2 | S | >16 | R |
| 43 | 1 | S | 2 | S |  |  |  |  | ≤0.5 | S | ≤0.5 | S | ≤0.5 | S | ≤1 | S | >16 | R |
| 44 | 0.5 | S | 1 | S | ≤2 | - |  |  | ≤0.5 | S |  |  | ≤0.25 | S |  |  | >32 | R |
| 45 | 1 | S | 2 | S | 1 | I | >8 | R | ≤0.5 | S | ≤0.5 | S | ≤0.5 | S | 2 | S | >16 | R |
| 46 | ≤0.12 | S | ≤0.25 | S |  |  |  |  | ≤0.5 | S | 1 | S | ≤0.5 | S | 4 | S | 0.5 | S |
| 47 | 0.5 | S | 1 | S | 2 | R | 8 | R | ≤0.5 | S |  |  | ≤0.5 | S | ≤1 | S | >16 | R |
| 48 | 0.5 | S | 1 | S |  |  |  |  | ≤0.5 | S | ≤0.5 | S | ≤0.25 | S |  |  | >32 | R |
| 49 | ≤0.06 | S | 0.25 | S | ≤0.5 | S |  |  | ≤0.5 | S | ≤0.5 | S | ≤0.25 | S | ≤1 | S | >32 | R |
| 50 | 1 | S | 1 | S | 1 | I | 8 | R | ≤0.5 | S |  |  | ≤0.5 | S | ≤1 | S | >16 | R |
| 51 | 0.25 | S | ≤0.25 | S | ≤0.5 | S | ≤0.5 | S | ≤0.5 | S |  |  | ≤0.5 | S | ≤1 | S | >16 | R |
| 52 | 1 | S | 1 | S | ≤0.5 | S |  |  | ≤0.5 | S | ≤0.5 | S | ≤0.5 | S | ≤1 | S | >16 | R |
| 53 | 1 | S | 2 | S | 1 | I | 8 | R | ≤0.5 | S |  |  | ≤0.5 | S | ≤1 | S | 16 | R |
| 54 | 0.5 | S | 1 | S | 1 | I | 8 | R | ≤0.5 | S |  |  | ≤0.5 | S | 2 | S | >16 | R |
| 55 | 0.5 | S | 1 | S | 1 | I | 8 | R | ≤0.5 | S |  |  | ≤0.5 | S | ≤1 | S | >16 | R |
| 56 | 4 | S | ≤8 | S | 2 | R | >8 | R | ≤0.5 | S |  |  | ≤0.5 | S | 2 | S | >16 | R |
| 57 | 1 | S | 1 | S | 2 | R | >8 | R | ≤0.5 | S |  |  | ≤0.5 | S | ≤1 | S | >16 | R |
| 58 | 1 | S | 1 | S | 1 | I | 8 | R | ≤0.5 | S |  |  | ≤0.5 | S | 2 | S | >16 | R |
| 59 | 0.5 | S | 0.5 | S | ≤0.5 | S | ≤0.5 | S | ≤0.5 | S |  |  | ≤0.5 | S | ≤1 | S | >16 | R |
| 60 | 0.5 | S | 1 | S | 1 | I | 8 | R | ≤0.5 | S |  |  | ≤0.5 | S | ≤1 | S | >16 | R |
| 61 | 4 | S | 4 | S | 2 | R | >8 | R | ≤0.5 | S |  |  | ≤0.5 | S | 2 | S | >16 | R |
| 62 | ≤0.12 | S | ≤0.25 | S | ≤0.5 | S | ≤0.5 | S | ≤0.5 | S |  |  | ≤0.5 | S | ≤1 | S | >16 | R |
| 63 | 1 | S | 1 | S | 1 | I | 8 | R | ≤0.5 | S |  |  | ≤0.5 | S | 2 | S | >16 | R |
| 64 | 1 | S | 1 | S | 1 | I | 8 | R | ≤0.5 | S |  |  | ≤0.5 | S | 2 | S | >16 | R |
| 65 | 1 | S | 2 | S | 1 | I | 8 | R | ≤0.5 | S | 2 | S | ≤0.5 | S | 2 | S | >16 | R |
| 66 | 0.5 | S | 1 | S | 1 | I | 8 | R | ≤0.5 | S |  |  | ≤0.5 | S | ≤1 | S | >16 | R |
| 67 | 0.5 | S | 1 | S | 2 | R | 8 | R | ≤0.5 | S |  |  | ≤0.5 | S | ≤1 | S | >16 | R |
| 68 | 1 | S | 1 | S | 1 | I |  |  | ≤0.5 | S | ≤0.5 | S | ≤0.25 | S | ≤1 | S | >32 | R |

Minimum inhibitory concentration (MIC) values were obtained using broth microdilution methods and applied breakpoints from the Clinical Laboratory Standards Institute (CLSI) M45.(1) The imipenem MIC for isolate No. 44 is reported here but excluded from Table 2 as it is uninterpretable according to CLSI breakpoints. Abbreviations: I, intermediate; Interp., interpretation; S, susceptible; R, resistant.

**Supplement 2:** STROBE checklist for cohort studies

When applicable to the present study, we adhered to the Strengthening the Reporting of Observational Studies in Epidemiology (STROBE) checklist for cohort studies.(2) Our study did not include separate reporting for exposed and unexposed groups as these groupings are not relevant to our study design.

|  | Item No | Recommendation |  | Item location in manuscript |  |
| --- | --- | --- | --- | --- | --- |
| **Title and abstract** | 1 | (*a*) Indicate the study’s design with a commonly used term in the title or the abstract |  | Abstract |  |
|  |  | (*b*) Provide in the abstract an informative and balanced summary of what was done and what was found |  |  |  |
| Introduction | | |  |  |  |
| Background/rationale | 2 | Explain the scientific background and rationale for the investigation being reported |  | Background |  |
| Objectives | 3 | State specific objectives, including any prespecified hypotheses |  | Background |  |
| Methods | | |  |  |  |
| Study design | 4 | Present key elements of study design early in the paper |  | Methods |  |
| Setting | 5 | Describe the setting, locations, and relevant dates, including periods of recruitment, exposure, follow-up, and data collection |  | Methods |  |
| Participants | 6 | (*a*) Give the eligibility criteria, and the sources and methods of selection of participants. Describe methods of follow-up |  | Methods |  |
|  |  | (*b*) For matched studies, give matching criteria and number of exposed and unexposed |  | Not applicable |  |
| Variables | 7 | Clearly define all outcomes, exposures, predictors, potential confounders, and effect modifiers. Give diagnostic criteria, if applicable |  | Methods |  |
| Data sources/ measurement | 8 | For each variable of interest, give sources of data and details of methods of assessment (measurement). Describe comparability of assessment methods if there is more than one group |  | Methods |  |
| Bias | 9 | Describe any efforts to address potential sources of bias |  | Methods |  |
| Study size | 10 | Explain how the study size was arrived at |  | Methods, Figure 1 |  |
| Quantitative variables | 11 | Explain how quantitative variables were handled in the analyses. If applicable, describe which groupings were chosen and why |  | Methods |  |
| Statistical methods | 12 | (*a*) Describe all statistical methods, including those used to control for confounding |  | Methods |  |
|  |  | (*b*) Describe any methods used to examine subgroups and interactions |  | Not applicable |  |
|  |  | (*c*) Explain how missing data were addressed |  | Methods |  |
|  |  | (*d*) If applicable, explain how loss to follow-up was addressed |  | Methods |  |
|  |  | (*e*) Describe any sensitivity analyses |  | Not applicable |  |
| Results | | |  |  |  |
| Participants | 13 | (a) Report numbers of individuals at each stage of study—eg numbers potentially eligible, examined for eligibility, confirmed eligible, included in the study, completing follow-up, and analysed |  | Results, Figure 1 |  |
|  |  | (b) Give reasons for non-participation at each stage |  | Figure 1 |  |
|  |  | (c) Consider use of a flow diagram |  | Figure 1 |  |
| Descriptive data | 14 | (a) Give characteristics of study participants (eg demographic, clinical, social) and information on exposures and potential confounders |  | Results |  |
|  |  | (b) Indicate number of participants with missing data for each variable of interest |  | Results |  |
|  |  | (c) Summarise follow-up time (eg, average and total amount) |  | Results |  |
| Outcome data | 15 | Report numbers of outcome events or summary measures over time |  | Results |  |
| Main results | 16 | (*a*) Give unadjusted estimates and, if applicable, confounder-adjusted estimates and their precision (eg, 95% confidence interval). Make clear which confounders were adjusted for and why they were included |  | Not applicable |  |
|  |  | (*b*) Report category boundaries when continuous variables were categorized |  | Results |  |
|  |  | (*c*) If relevant, consider translating estimates of relative risk into absolute risk for a meaningful time period |  | Not applicable |  |
| Other analyses | 17 | Report other analyses done—eg analyses of subgroups and interactions, and sensitivity analyses |  | Not applicable |  |
| Discussion | | |  |  |  |
| Key results | 18 | Summarise key results with reference to study objectives |  | Discussion |  |
| Limitations | 19 | Discuss limitations of the study, taking into account sources of potential bias or imprecision. Discuss both direction and magnitude of any potential bias |  | Discussion |  |
| Interpretation | 20 | Give a cautious overall interpretation of results considering objectives, limitations, multiplicity of analyses, results from similar studies, and other relevant evidence |  | Discussion |  |
| Generalisability | 21 | Discuss the generalisability (external validity) of the study results |  | Discussion |  |
| Other information | | |  |  |  |
| Funding | 22 | Give the source of funding and the role of the funders for the present study and, if applicable, for the original study on which the present article is based |  | Funding statement |  |

References

1. CLSI. Methods for Antimicrobial Dilution and Disk Susceptibility Testing of Infrequently Isolated or Fastidious Bacteria. 3rd ed. Wayne, PA: Clinical and Laboratory Standards Institute; 2015.

2. von Elm E, Altman DG, Egger M, et al. The Strengthening the Reporting of Observational Studies in Epidemiology (STROBE) statement: guidelines for reporting observational studies. Ann Intern Med. 2007;147(8):573-7.
